# Co-infection with *Schistosoma mansoni* and *Helicobacter pylori*: epidemiological patterns and implications for integrated control

**DOI:** 10.1016/j.parepi.2026.e00535

**Published:** 2026-08-19

**Authors:** Ashraf Fawzy Mosa Ahmed, Hala Shehata Ali, Nabeel H. Alhussainy, Hany M. El-Wahsh, Bander Hasan Saleh, Turki Asiri, Karem Ibrahem, Fadi Baakdah, Haifaa A. Mahjoub, Wesam K. Bakhsh, Ala A. Azhari, Hatoon A. Niyazi, Doaa Naguib

**Affiliations:** a Parasitology Department, Medical Research Institute, Alexandria University, Alexandria, Egypt; b Department of Clinical Microbiology and Immunology, Faculty of Medicine, King Abdulaziz University, Jeddah 21589, Saudi Arabia; c Marine Biology Department, Faculty of Marine Sciences, King Abdulaziz University, Jeddah 21589, Saudi Arabia; d Department of Microbiology and Parasitology, Faculty of Medicine, King Abdulaziz University, Rabigh, Saudi Arabia; e Department of Medical Laboratory Sciences, Faculty of Applied Medical Sciences, King Abdulaziz University, Jeddah 21589, Saudi Arabia; f Special Infectious Agents Unit, King Fahd Medical Research Center, King Abdulaziz University, Jeddah 21589, Saudi Arabia; g Department of Biological Sciences, College of Science & Arts, King Abdulaziz University, Rabigh 21911, Saudi Arabia; h Department of Basic Medical Sciences, College of Medicine, University of Jeddah, Jeddah, Saudi Arabia; i Department of Hygiene and Zoonoses, Faculty of Veterinary Medicine, Mansoura University, Mansoura 35516, Egypt

**Keywords:** *Schistosoma mansoni*, *Helicobacter pylori*, Co-infection, Occupational exposure, Neglected tropical disease, Hematological parameters, Egypt

## Abstract

Co-infections with *Schistosoma mansoni* and *Helicobacter pylori* are frequently reported in low-resource endemic settings; however, their combined systemic and hematological effects in true co-endemic populations remain incompletely characterized. While *S. mansoni* transmission is primarily driven by exposure to agricultural water, *H. pylori* reflects persistent household and sanitation-related transmission, highlighting distinct yet overlapping epidemiological pathways. This study aimed to assess the association between occupational freshwater exposure and *S. mansoni* infection and to evaluate the clinical, hematological, and biochemical profiles associated with *S. mansoni* and *H. pylori* mono- and co-infections in a rural Egyptian population. A community-based, cross-sectional study was conducted in a rural agricultural village in Egypt. Participants were stratified into four groups: *S. mansoni* mono-infection (Gp1), *S. mansoni-H. pylori* co-infection (Gp2), *H. pylori* mono-infection (Gp3), and uninfected controls (Gp4). Clinical assessment, stool microscopy using the Kato-Katz thick smear technique, *H. pylori* coproantigen ELISA, complete blood counts, and serum biochemical analyses were performed. Comparative statistical analyses were used to evaluate group differences and associated risk factors.

Occupational exposure (farm work) was strongly associated with *S. mansoni* infection (*P* < 0.001), reinforcing its role as a key transmission driver in rural endemic settings. Gastrointestinal symptoms were largely non-specific, although hematochezia occurred exclusively among individuals infected with *S. mansoni*. Co-infection did not significantly influence *H. pylori* antigen levels (*P* = 0.190), indicating limited biological interaction between the pathogens. Systemic alterations were predominantly driven by *S. mansoni*, including thrombocytopenia and marked eosinophilia (*P* < 0.001), alongside elevated serum globulin levels (*P* = 0.037), suggesting sustained immune activation. Other biochemical parameters showed no significant differences.

In endemic rural settings, co-infection with *S. mansoni* and *H. pylori* does not demonstrate synergistic systemic effects but rather reflects overlapping transmission pathways with distinct epidemiological determinants. These findings suggest that integrated screening strategies may offer limited additional clinical benefit beyond pathogen-specific management, while still supporting coordinated control efforts targeting shared risk environments.

## 1 Introduction

Schistosomiasis remains a major neglected tropical disease affecting human populations worldwide, with more than 250 million people requiring preventive treatment, according to the World Health Organization (WHO, 2026). Despite sustained control efforts, the disease continues to impose a significant public health burden in endemic regions (Cissé et al., 2026; Inobaya et al., 2014). Among human-infecting species, *S. mansoni* infection is predominant in parts of Africa and the Middle East, including Egypt, where transmission persists in focal rural areas of the Nile Delta (Barakat, 2013). Within this region, El-Behira Governorate, including Abou-Homos district, is recognized as a focal endemic area for *S. mansoni* based on national surveillance reports and multiple epidemiological studies, reflecting sustained transmission in agricultural communities (Ahmed et al., 2021; Barakat, 2013). Chronic infection is associated with intestinal pathology, eosinophilia, and systemic hematological alterations that reflect ongoing host-parasite interactions (Bisetegn et al., 2022; Gonçalves-Silva et al., 2022).

*H. pylori* infection is another highly prevalent infection in low-resource settings, characterized by lifelong gastric colonization and linked to gastritis, peptic ulcer disease, and gastric cancer (Kalenić et al., 2002). In addition to its gastrointestinal effects, *H. pylori* has been associated with systemic hematological changes, including alterations in platelet counts and iron metabolism (Sağlam and Civan, 2023). In Egypt, *H. pylori* infection remains highly prevalent across both adult and pediatric populations (Alboraie et al., 2026; Bassily et al., 1999).

The transmission pathways of these two infections differ substantially. *S. mansoni* is primarily associated with environmental and occupational exposure to contaminated freshwater, particularly in agricultural settings (Hailegebriel et al., 2022; Walz et al., 2015), whereas *H. pylori* transmission is closely linked to household conditions, sanitation, and interpersonal contact (Fowora et al., 2025). These distinct yet overlapping epidemiological patterns create conditions in which co-infections are common in rural endemic communities. Although both pathogens independently influence hematological and clinical parameters, data on their combined effects in human populations remain limited (Afful et al., 2024; Muttiah et al., 2025). Existing studies have largely focused on infection prevalence, with relatively few community-based investigations examining whether co-infection modifies systemic profiles or disease expression (Ahmed et al., 2021; Fowora et al., 2025). Understanding these interactions is important in endemic settings, where overlapping infections may complicate clinical assessment and influence disease management strategies. This study aimed to investigate the association between occupational exposure and *S. mansoni* infection and to evaluate the clinical, hematological, and biochemical profiles associated with *S. mansoni* and *H. pylori* mono- and co-infections in a rural Egyptian population. By integrating epidemiological risk factors with laboratory findings, this work seeks to determine whether co-infection exerts additive or distinct systemic effects and to assess its potential relevance for integrated disease control approaches in endemic settings.

## 2 Material and methods

### 2.1 Study design and setting

A community-based cross-sectional study was conducted in El-Roda village, Abou-Homos district, El-Behira Governorate, Egypt. The village has an estimated population of approximately 10,000 residents, according to local administrative records. The area is a rural agricultural setting with extensive reliance on irrigation canals and freshwater sources, posing a high risk of waterborne parasitic infections, particularly *S. mansoni*. The site was selected based on its documented endemicity and sustained occupational exposure among farming communities.

Participants were recruited through local healthcare units in coordination with public health authorities to ensure representation across varying levels of environmental and occupational exposure. A non-probability sampling approach was applied to include both high-risk agricultural workers and non-exposed residents. The study was conducted and reported in accordance with STROBE guidelines for cross-sectional studies (Cuschieri, 2019).

### 2.2 Ethical approval

The study protocol was approved by the Ethics Committee of the Medical Research Institute, Alexandria University (IORG No. 0008812), with additional authorization from the Egyptian Ministry of Health and Population. Informed consent was obtained from all participants or their legal guardians, and assent was obtained from underage participants. All procedures were conducted in accordance with the Declaration of Helsinki. Participants diagnosed with *S. mansoni* or *H. pylori* infections received appropriate treatment in accordance with national guidelines.

### 2.3 Study population and grouping

A total of 200 participants (80 males and 120 females; age range: 12–40 years) were enrolled and equally allocated into four groups (*n* = 50 each) based on laboratory-confirmed infection status:

• Gp1: Patients infected with *S. mansoni* only, as determined by the Kato-Katz thick smear technique.
• Gp2: Patients co-infected with *S. mansoni* (Kato-Katz) and *H. pylori* (stool antigen ELISA).
• Gp3: Patients infected with *H. pylori* only, confirmed by stool antigen ELISA.
• Gp4: Uninfected controls, negative by both Kato-Katz and *H. pylori* ELISA.

Eligibility criteria included permanent residence in the study area and confirmed infection status. Individuals with other parasitic infections, chronic systemic diseases (e.g., diabetes, hepatic or renal disorders, or immunodeficiency), or recent use of antibiotics or antiparasitic drugs were excluded. Sample size calculation indicated a minimum of 30 participants per group (α = 0.05, power = 80%, effect size f = 0.25); however, 50 participants per group were included to account for field variability.

### 2.4 Data collection

Demographic and clinical data were collected using a structured questionnaire that included age, sex, occupational exposure (farm work and freshwater contact), gastrointestinal symptoms, and hygiene-related practices. Farm work exposure was recorded as a binary variable (yes/no). Environmental and hygiene-related factors were also assessed. Socioeconomic variables such as income and education were not collected because the study focused on targeted epidemiological and hematological assessments rather than a comprehensive socioeconomic survey.

### 2.5 Sample collection and processing

Stool and blood samples were collected from all participants under standardized and aseptic conditions to ensure consistency and minimize pre-analytical variability. A single fresh stool sample was obtained from each participant in a sterile, labeled container and processed promptly. Each sample was divided into three aliquots: the first was examined using the formalin-ether sedimentation technique to exclude other intestinal parasitic infections; the second was processed using the Kato-Katz thick smear technique for detection and quantification of *S. mansoni* eggs, expressed as eggs per gram (EPG); and the third was stored at −20 °C for *H. pylori* stool antigen detection using a commercial ELISA kit (Egyptian Trade Co., Cairo, Egypt) following manufacturer instructions to ensure reliable antigen preservation and detection (Nulty et al., 2021). Approximately 5 mL of venous blood was collected from each participant using sterile techniques. One milliliter of whole blood was transferred into Ethylenediaminetetraacetic acid (EDTA) tubes for hematological assessment, including complete blood count (CBC) and derived inflammatory indices. The remaining blood was allowed to clot and subsequently centrifuged at 3000 ×*g* for 10 min to obtain serum. The separated serum was aliquoted and stored at −20 °C until biochemical analyses were performed to measure total protein, albumin, globulin, and ferritin concentrations. All laboratory procedures were performed under standardized conditions to ensure reproducibility across study groups.

### 2.6 Parasitological and microbiological diagnosis

Infection with *S. mansoni* was confirmed by microscopic identification of characteristic ova in stool samples using the Kato-Katz thick smear technique, a standardized quantitative method for diagnosing intestinal schistosomiasis. Two Kato-Katz thick smear slides were prepared and examined for each stool sample to increase diagnostic sensitivity and ensure reliable quantification of infection intensity. Egg counts were expressed as eggs per gram (EPG) of stool, and geometric mean egg counts (GMEC) were calculated for each study group (Colley et al., 2014). All slides were prepared and examined by trained laboratory personnel to ensure diagnostic accuracy and minimize inter-observer variability. *H. pylori* infection was detected using a stool antigen ELISA, a non-invasive and widely accepted method for identifying active infection. Stool samples were processed according to the manufacturer's instructions with strict adherence to incubation times, reagent preparation, and quality-control procedures. Optical density values were measured using a microplate reader, and results were classified as positive or negative based on predefined cutoff values validated against established diagnostic criteria. (Malfertheiner et al., 2022). This standardized approach ensured consistent and accurate detection of active *H. pylori* infection across all study participants.

### 2.7 Hematological and biochemical analyses

Hematological parameters were measured using an automated hematology analyzer (Sysmex XN-1000, Japan), which provided standardized measurements of complete blood count (CBC) indices, including hemoglobin concentration, red blood cell (RBC) count, hematocrit, total leukocyte count, differential leukocyte counts (neutrophils, lymphocytes, monocytes, and eosinophils), and platelet count (Seo et al., 2015). To assess systemic inflammatory status, the neutrophil-to-lymphocyte ratio (NLR) and platelet-to-lymphocyte ratio (PLR) were calculated as derived hematological indices, both of which are established markers of systemic inflammation and immune activation. Biochemical analyses were performed on serum samples using standardized laboratory methods. Serum total protein and albumin concentrations were determined by automated colorimetric assays, while globulin concentration was calculated by subtracting albumin from total protein (Zheng et al., 2017). In addition, serum ferritin concentrations were quantified using a commercial enzyme immunoassay kit (Abbott Diagnostics, USA) according to the manufacturer's instructions, with strict adherence to calibration, incubation, and quality-control procedures to ensure accuracy and reproducibility of results (Punnonen et al., 1997).

### 2.8 Statistical analysis

Data were analyzed using IBM SPSS Statistics version 20. Normality was assessed using the Shapiro-Wilk test. Parametric data were analyzed using one-way ANOVA with Tukey's post hoc test, while non-parametric data were analyzed using the Kruskal–Wallis test. Categorical variables were compared using the Chi-square test. Multivariate regression analysis was performed to identify independent predictors. Statistical significance was set at *p* < 0.05.

## 3 Results

### 3.1 Association between farm work and infection status

Farm work was strongly associated with *S. mansoni* infection. All participants in Gp1 and Gp2 reported agricultural activities (100% each), compared with 16% in the *H. pylori* mono-infection group (Gp3) and 44% in controls (Gp4). This difference was highly significant (χ^2^ = 58.154, *P* < 0.001), indicating that occupational freshwater exposure is a major determinant of *S. mansoni* infection, whereas no clear association was observed with *H. pylori* infection (Table 1).

**Table 1:** Distribution of farm-working status among the studied groups.

Farm working	Studied groups								χ^2^	P
	Gp1		Gp2		Gp3		Gp4			
	No.	%	No.	%	No.	%	No.	%		
Yes	50	100	50	100	8	16	22	44	58.154	<0.001⁎
No	0	0	0	0	42	84	28	56		
Total	50	100	50	100	50	100	50	100		

Gp1: Patients infected with *S. mansoni* only.

Gp2: Patients co-infected with *S. mansoni* and *H. pylori*.

Gp3: Patients infected with *H. pylori* only.

Gp4: Uninfected controls.

χ^2^: Chi-square test.

⁎ Statistically significant at *P* < 0.05.

### 3.2 Clinical manifestations

Clinical symptoms were generally non-specific across groups. Abdominal pain was the most frequently reported symptom (96–100% in all groups), followed by variable gastrointestinal complaints. Hematochezia was exclusively observed in *S. mansoni*-infected individuals (Gp1: 8%, Gp2: 4%) and was absent in *H. pylori* mono-infection and control groups. Other symptoms, including vomiting and diarrhea, showed no group-specific pattern (Table 2).

**Table 2:** Frequency and percentage of clinical manifestations among the three patient groups.

Clinical manifestation	Study patient groups					
	Gp1 (*n* = 50)		Gp2 (*n* = 50)		Gp3 (*n* = 50)	
	No.	%	No.	%	No.	%
Blood in stool	4	8	2	4	0	0
Dyspepsia	0	0	0	0	0	0
Vomiting	14	28	32	64	24	48
Abdominal pain	48	96	50	100	50	100
Diarrhea	16	32	24	48	32	64
Fever	2	4	0	0	2	4
Fatigue	10	20	6	12	16	32

Gp1: Patients infected with *S. mansoni* only.

Gp2: Patients co-infected with *S. mansoni* and *H. pylori*.

Gp3: Patients infected with *H. pylori* only.

### 3.3 Intensity of *Schistosoma mansoni* infection

**Fig. 1:**
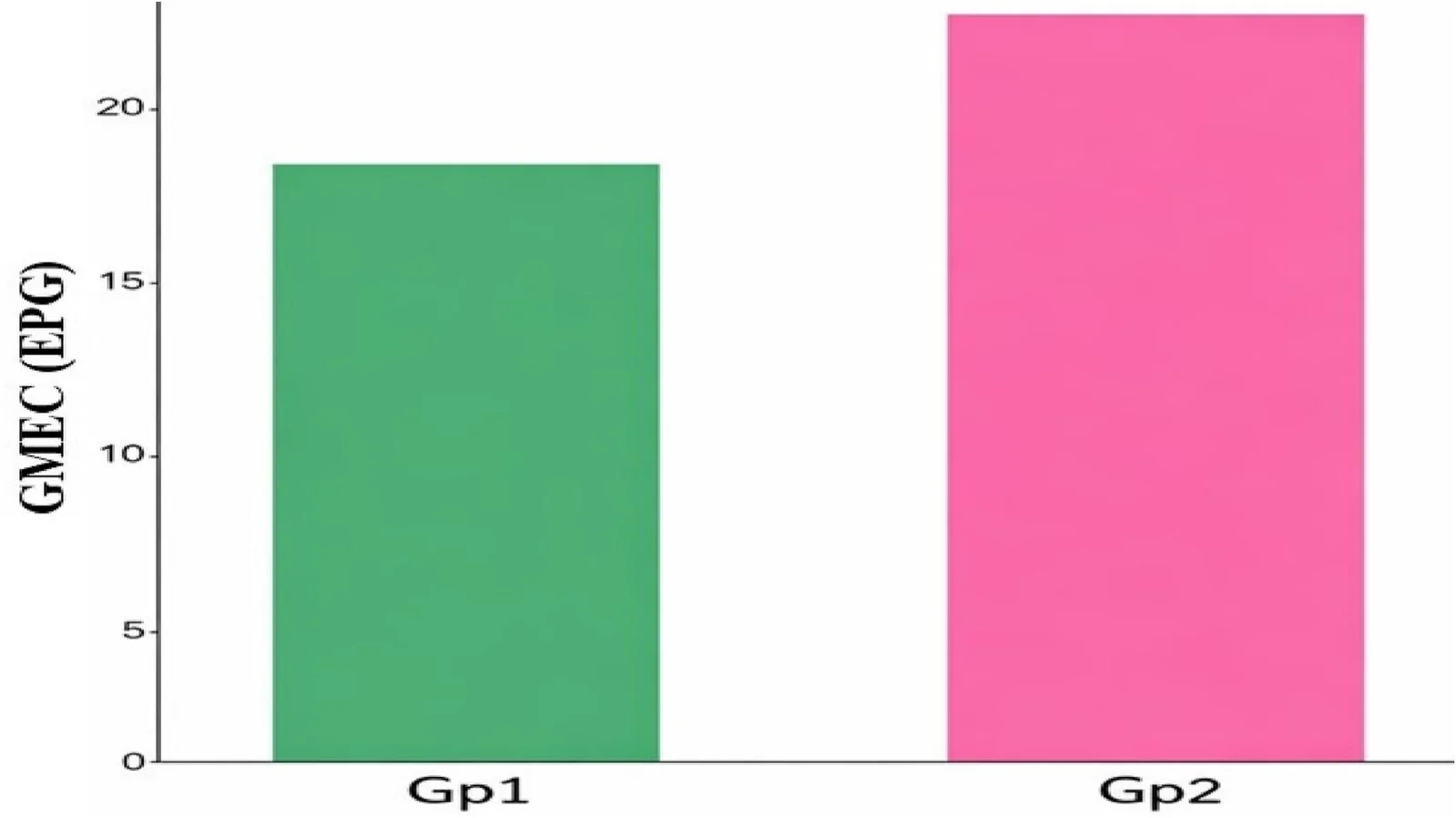
Geometric mean egg count (GMEC; eggs per gram of feces [EPG]) in the two *Schistosoma mansoni*-infected groups. Gp1 (patients infected with *S. mansoni* only) and Gp2 (patients co-infected with *S. mansoni* and *H. pylori*).

Infection intensity was predominantly low in both schistosomiasis groups. The geometric mean egg counts were 18.04 EPG in Gp1 and 22.34 EPG in Gp2 (Fig. 1). Most cases fell within the low-intensity category (1–99 EPG), with 49 cases in Gp1 and 50 in Gp2. Only one moderate infection was detected (Gp1), and none were recorded in the co-infected group.

### 3.4 *Helicobacter pylori* coproantigen levels

**Fig. 2:**
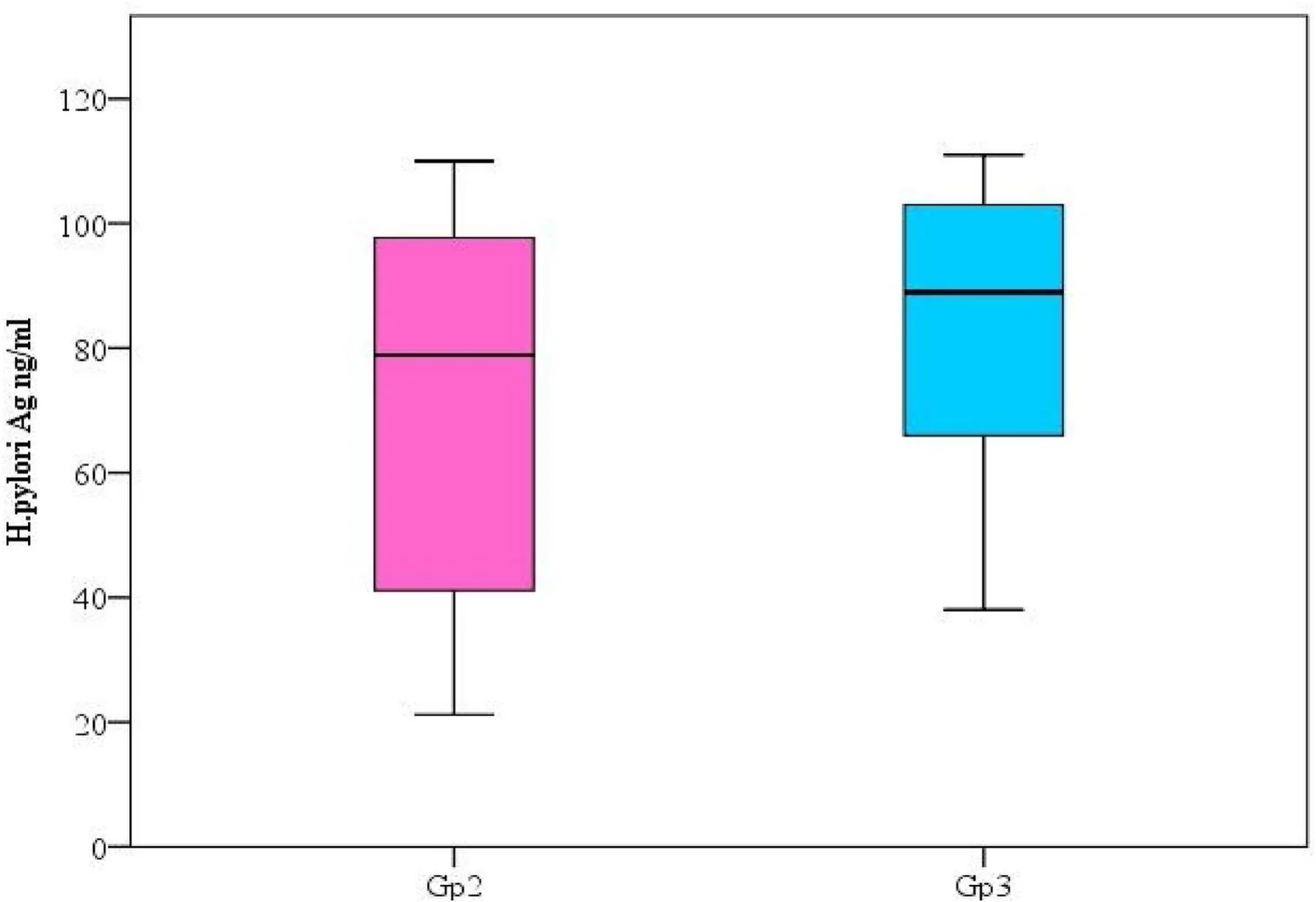
*H. pylori* antigen levels in Gp2 and Gp3. Distribution of *Helicobacter pylori* antigen concentrations (ng/ml) in Gp2 (patients co-infected with *S. mansoni* and *H. pylori)* and Gp3 (patients infected with *H. pylori* only), showing median values, interquartile ranges, and overall variability between the two groups.

Stool antigen concentrations of *H. pylori* were comparable between infected groups. Median values were slightly higher in Gp3 (89.0 ng/mL) compared with Gp2 (78.87 ng/mL), with overlapping distributions (20–110 ng/mL). However, the difference was not statistically significant (*P* = 0.190), indicating that *S. mansoni* co-infection did not affect *H. pylori* antigen burden (Fig. 2).

### 3.5 Hematological parameters

No significant differences were observed in hemoglobin, RBC count, hematocrit, or red cell indices across groups. Hemoglobin levels ranged from 12.07 ± 1.36 g/dL (Gp1) to 12.82 ± 1.13 g/dL (Gp4), with no statistical significance (*P* > 0.05). Similarly, MCV, MCH, and MCHC remained stable across all groups (Table 3). Overall, infection status had no measurable impact on basic hematological parameters.

**Table 3:** Hematological parameters across the studied groups.

Variables	Gp1 (n = 50)	Gp2 (n = 50)	Gp3 (n = 50)	Gp4 (n = 50)	Test of sig. (F)	P
Hb (g/dl)	12.07 ± 1.36	12.14 ± 1.44	12.61 ± 1.35	12.82 ± 1.13	0.726	0.536
RBCs (10^6^cell/ul)	4.32 ± 0.38	5.49 ± 0.37	4.52 ± 0.39	4.39 ± 0.39	1.654	0.182
HCT (%)	39.87 ± 4.51	40.06 ± 4.77	41.62 ± 4.54	40.66 ± 4.86	0.709	0.549
MCV (fl)	92.02 ± 3.91	92.81 ± 3.48	91.98 ± 3.73	92.32 ± 4.07	0.255	0.858
MCH (pg)	27.89 ± 1.16	28.13 ± 1.01	27.80 ± 1.17	27.94 ± 1.21	0.374	0.772
MCHC (%)	30.56 ± 0.44	31.02 ± 0.76	30.98 ± 0.87	30.45 ± 0.44	4.833	0.051

Gp1: Patients infected with *S. mansoni* only.

Gp2: Patients co-infected with *S. mansoni* and *H. pylori.*

Gp3: Patients infected with *H. pylori* only.

Gp4: Uninfected controls.

Values are expressed as mean ± SD.

(Hb) hemoglobin, (RBCs) red blood cell count, (HCT) hematocrit, (MCV) mean corpuscular volume, (MCH) mean corpuscular hemoglobin, and (MCHC) mean corpuscular hemoglobin concentration.

F: F-value for one-way ANOVA; post hoc comparisons were performed using Tukey's test.

P: *P*-value for comparison among the studied groups.

### 3.6 Platelet count variations

**Fig. 3:**
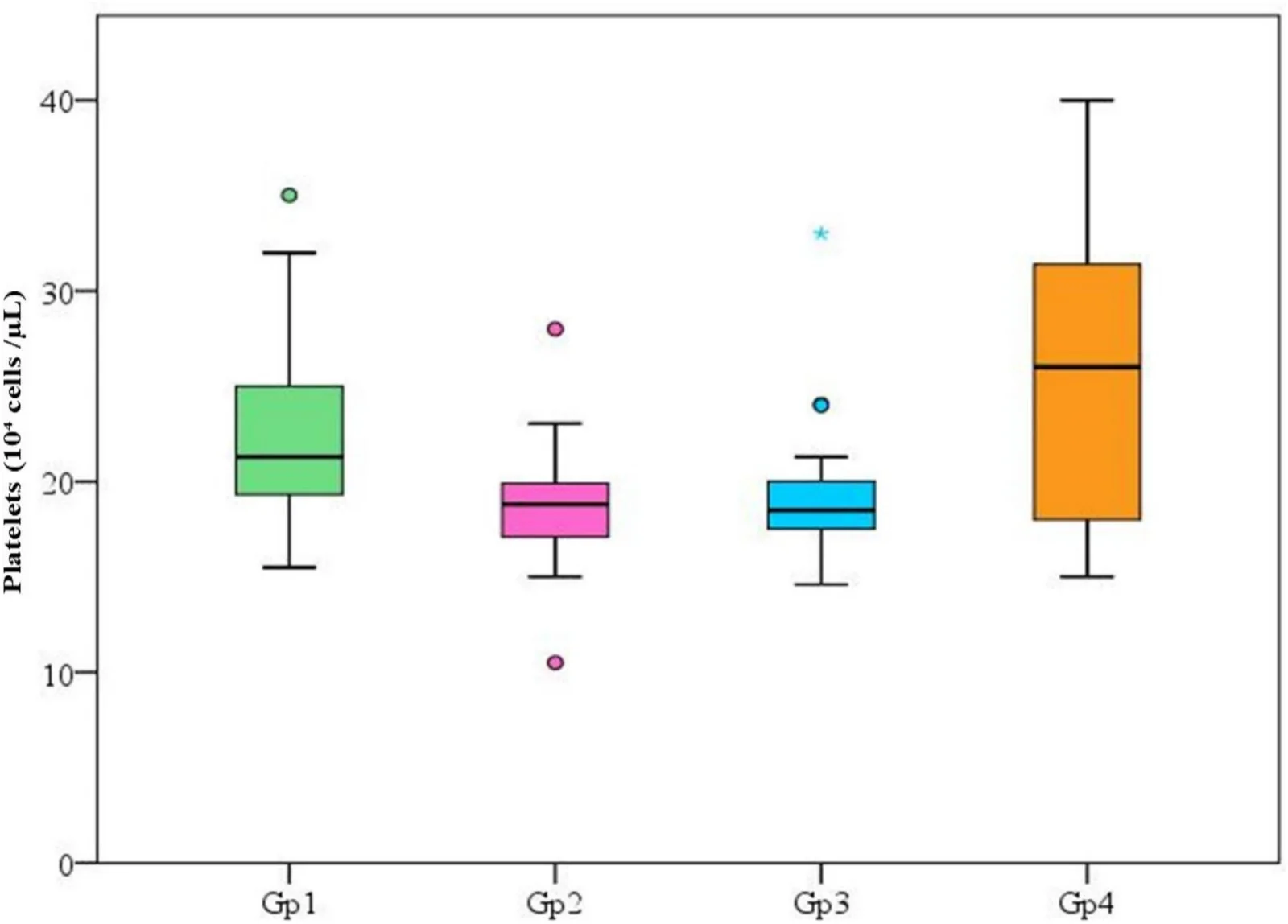
Distribution of platelet counts (10^4^ cells/μL) among the four studied groups: Gp1 (patients infected with *S. mansoni* only), Gp2 (patients co-infected with *S. mansoni* and *H. pylori*), Gp3 (patients infected with *H. pylori* only), and Gp4 (uninfected controls). Data are presented as median values with interquartile ranges (IQR), with whiskers indicating overall variability and points representing outliers. A significant reduction in platelet counts was observed in all infected groups compared with controls (*P* < 0.001), highlighting an infection-associated thrombocytopenic effect.

Platelet counts differed significantly among groups (*P* < 0.001). All infected groups showed reduced platelet counts compared with controls, with the lowest values observed in the co-infection group. In contrast, controls (Gp4) exhibited the highest platelet levels, suggesting an infection-associated thrombocytopenic pattern (Fig. 3).

### 3.7 Leukocyte differential counts

**Fig. 4:**
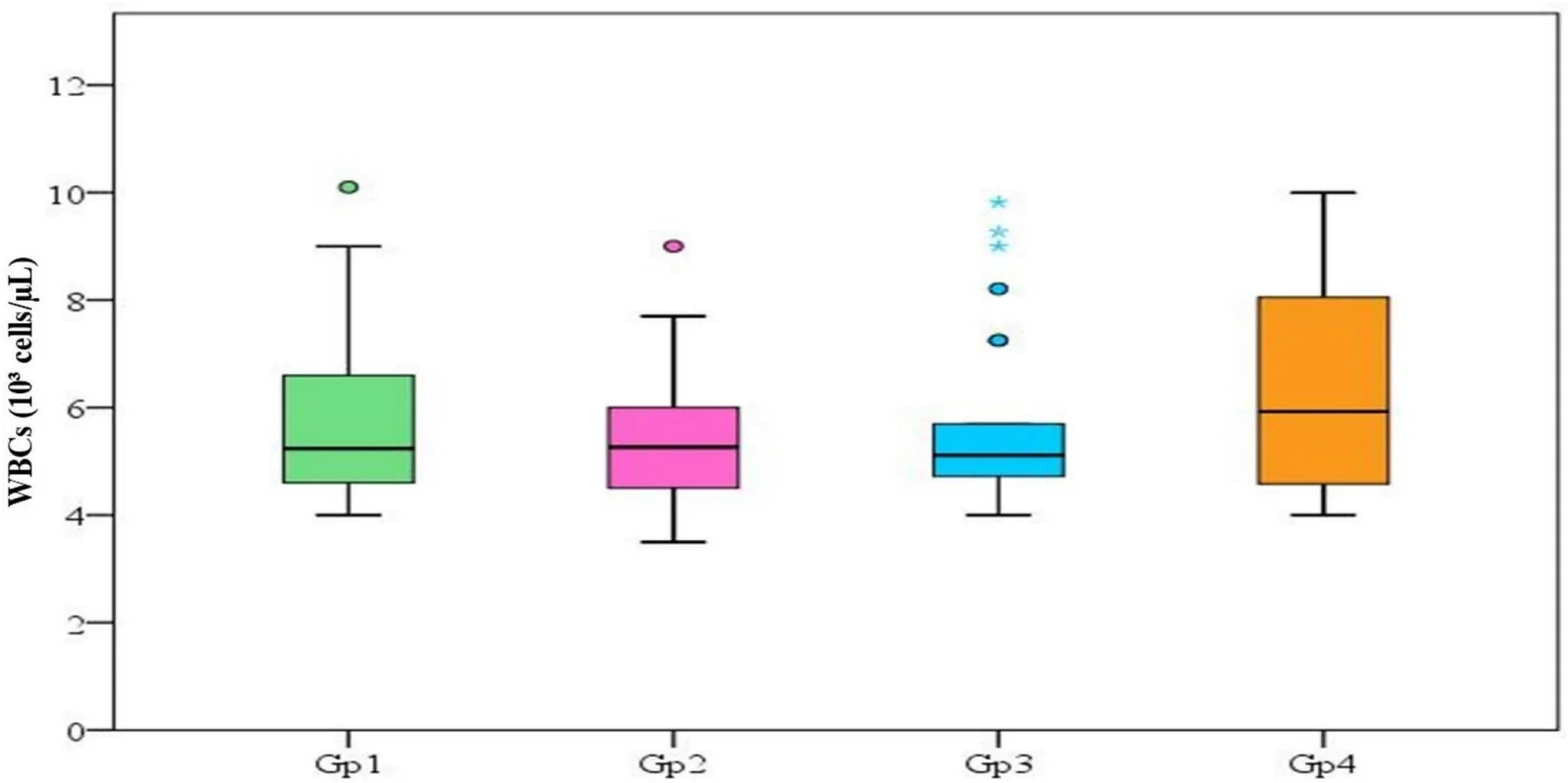
The distribution of total white blood cell (WBC) counts (10^3^ cells/μL) across four studied groups: Gp1 (patients infected with *S. mansoni* only), Gp2 (patients co-infected with *S. mansoni* and *H. pylori*), Gp3 (patients infected with *H. pylori* only), and Gp4 (uninfected controls). The data are presented as medians with interquartile ranges (IQR), and individual points represent outliers. Overall, WBC counts are similar across groups, indicating that infection status has a limited impact. However, the variability within groups reflects diverse host immune responses.

Significant variations were observed in leukocyte subsets. Neutrophil percentages differed across groups (*P* = 0.004), with higher values in Gp3. Eosinophil counts showed a marked and highly significant increase in *S. mansoni*-infected groups (Gp1 and Gp2) compared with other groups (*P* < 0.001), representing the most prominent hematological signature of schistosomiasis. In contrast, lymphocytes, monocytes, and total WBC counts showed no significant differences (*P* > 0.05) (Fig. 4, Table 4).

**Table 4:** Leukocyte differential counts and total white blood cell (WBC) levels across the studied groups.

Parameters	Gp1 (n = 50)	Gp2 (n = 50)	Gp3 (n = 50)	Gp4 (n = 50)	Test of sig. (F)	P
Neutrophils (%)					4.72	0.004⁎
Min. – Max.	39–70	50–63	48–70	44–72		
Mean ± SD.	55.95 ± 6.63^b^	59.66 ± 3.35 ^a^	60.72 ± 4.74 ^a^	56.82 ± 6.14^b^		
Median	58	55.85	60	60		
Lymphocytes (%)					0.36	0.781
Min. – Max.	20–40	21–33	20–37	20–40		
Mean ± SD.	26.81 ± 5.56	25.91 ± 3.28	25.76 ± 4.26	26.83 ± 5.52		
Median	25.1	25.7	25	25		
Monocytes (%)					0.42	0.735
Min. – Max.	4–13	3–12	6–11	4–13		
Mean ± SD.	9.19–2.08	8.85–2.16	9.3–1.55	9.48–2.2		
Median	9.2	9.5	10	9.9		
Eosinophils (%)					59.54	<0.001⁎
Min. – Max.	6–10	7–11	2–8	1–8		
Mean ± SD.	8.21 ± 1.22 ^a, b^	8.37 ± 1.32 ^c, d^	4.22 ± 1.68 ^b, c^	4.27 ± 1.76 ^a, d^		
Median	8	8.1	4.1	4		
WBCs (10^3^cell/ μL)					^KW^χ^2^=1.53	0.674
Min. – Max.	4–10.10	3.50–9	4–9.82	4–10		
Mean ± SD.	5.71 ± 1.57	5.43 ± 1.28	5.71 ± 1.72	6.29 ± 1.98		
Median	5.24	5.26	5.11	5.93		

Gp1: Patients infected with *S. mansoni* only.

Gp2: Patients co-infected with *S. mansoni* and *H. pylori.*

Gp3: Patients infected with *H. pylori* only.

Gp4: Uninfected controls.

Data are presented as range (minimum–maximum), mean ± standard deviation (SD), and median values.

Different superscript letters indicate statistically significant differences between groups.

F: F-value for one-way ANOVA; post hoc comparisons were performed using Tukey's test.

KWχ^2^: Chi-square value for Kruskal-Wallis test; pairwise comparisons were performed using the Mann-Whitney *U* test.

P: *P*-value for comparison among the studied groups.

⁎ Statistically significant at *P* < 0.05.

### 3.8 Serum protein profile

Serum total protein and albumin levels did not differ significantly among groups (*P* = 0.419 and *P* = 0.505, respectively). However, globulin levels showed a modest but significant variation (*P* = 0.037), with higher values in *S. mansoni*-infected groups (Gp1 and Gp2), suggesting infection-associated immune activation. Co-infection did not further amplify this effect (Table 5).

**Table 5:** Serum protein profile (total protein, globulin, and albumin) among the studied groups.

Serum protein profile	Gp1 (*n* = 50)	Gp2 (*n* = 50)	Gp3 (*n* = 50)	Gp4 (n = 50)	F	P
Total protein (g/dl)					0.952	0.419
Min. – Max.	5.52–8.30	5.50–8.38	5.38–8.53	5.93–9.44		
Mean ± SD.	7.16 ± 0.66	7.19 ± 0.62	6.92 ± 0.67	6.99 ± 0.74		
Median	7.18	7.11	6.95	6.99		
Globulin (g/dl)					2.942	0.037⁎
Min. – Max.	2.10–4.51	1.07–4.25	0.96–5.29	1.92–5.02		
Mean ± SD.	3.20 ± 0.61^a^	3.03 ± 0.74^a^	2.64 ± 0.81^b^	2.85 ± 0.62^ab^		
Median	3.12	3.15	2.41	2.8		
Albumin (g/dl)					0.786	0.505
Min. – Max.	3.19–4.74	3.08–5.05	2.37–5.34	2.17–4.97		
Mean ± SD.	3.98 ± 0.47	4.16 ± 0.59	4.20 ± 0.61	4.15 ± 0.54		
Median	3.94	4.25	4.35	4.13		

Gp1: Patients infected with *S. mansoni* only.

Gp2: Patients co-infected with *S. mansoni* and *H. pylori.*

Gp3: Patients infected with *H. pylori* only.

Gp4: Uninfected controls.

Data are presented as range (minimum–maximum), mean ± standard deviation (SD), and median values.

F: F-value from one-way ANOVA. Post-hoc analysis: Significant differences between groups were assessed using Tukey's post-hoc test.

Different superscript letters indicate statistically significant differences between groups.

P: *P*-value for comparison among the studied groups.

⁎ Statistically significant at *P* < 0.05.

### 3.9 Serum ferritin levels

Serum ferritin levels showed wide inter-individual variation with no statistically significant differences between groups (*P* = 0.252). Although slightly higher median values were observed in the *H. pylori* mono-infection group (Gp3) and lower values in Gp1, distributions largely overlapped across all groups, indicating no consistent effect of infection status on iron stores (Table 6).

**Table 6:** Serum ferritin levels in the studied groups.

Ferritin level (ng/ml)	studied groups				^KW^χ^2^	P
	Gp1 (*n* = 50)	Gp2 (*n* = 50)	Gp3 (*n* = 50)	Gp4 (*n* = 50)		
Min. – Max.	3–350	2.5–280	13–325	5–155	4.088	0.252
Mean ± SD.	59.66 ± 87.77	45.93 ± 58.20	66.36 ± 74.47	48.87 ± 38.65		
Median	28	31	48	37.69		

Gp1: Patients infected with *S. mansoni* only.

Gp2: Patients co-infected with *S. mansoni* and *H. pylori.*

Gp3: Patients infected with *H. pylori* only.

Gp4: Parasite-free control subjects.

KW^2^: Chi-square value from the Kruskal–Wallis test used for comparison among the four groups. Data are presented as ranges (minimum–maximum), mean ± standard deviation (SD), and median values.

P: *P*-value for comparison among the studied groups.

## 4 Discussion

The present study provides an integrated evaluation of the epidemiological, clinical, hematological, and biochemical profiles associated with *S. mansoni* and *H. pylori* mono- and co-infections in a rural endemic setting. Overall, the findings demonstrate that both pathogens coexist in the same population but exert largely independent effects, with *S. mansoni* acting as the principal driver of systemic immuno-hematological alterations.

A strong association was observed between farm work and *S. mansoni* infection, reinforcing the central role of occupational freshwater exposure in transmission dynamics. This aligns with the well-established ecology of schistosomiasis, where human contact with contaminated irrigation systems sustains the parasite lifecycle through intermediate snail hosts (Bartlett et al., 2023; Tavakoli Pirzaman et al., 2024). In contrast, the weak association between farm work and *H. pylori* infection underscores its predominantly fecal-oral mode of transmission, which is more strongly influenced by household hygiene and living conditions than by occupational exposure (Schuppler, 2025).

Clinically, abdominal pain was the predominant symptom across all infected groups, underscoring its nonspecific nature in gastrointestinal infections. However, the exclusive occurrence of blood in stool among *S. mansoni*-infected individuals reflects the well-established pathology of intestinal schistosomiasis. Parasite eggs elicit granulomatous inflammation in the intestinal wall, leading to mucosal injury and bleeding (Hong et al., 2024). This symptom pattern provides a valuable clinical clue for distinguishing schistosomiasis from other gastrointestinal infections in endemic settings (Ponzo et al., 2024).

The predominance of low-intensity *S. mansoni* infection in both mono- and co-infected groups represents another important finding. The absence of significant differences in parasite burden between groups indicates that *H. pylori* co-infection does not meaningfully alter helminth intensity. This observation is consistent with previous evidence showing that host-parasite interactions in schistosomiasis are largely driven by infection intensity, with more pronounced systemic effects typically associated with moderate or heavy worm burdens (Kamau et al., 2021). Moreover, recent studies suggest that schistosome infections can modulate immune responses without necessarily affecting pathogen load, particularly in low-intensity infections (Ahmed et al., 2021; Costain et al., 2022). Similarly, *H. pylori* antigen levels were not significantly influenced by co-infection, supporting the concept of immunological compartmentalization. While schistosomiasis elicits a strong Th2-skewed immune response driven by egg antigens and associated cytokines (Abdel Aziz et al., 2022), *H. pylori* infection is typically characterized by Th1/Th17-mediated gastric inflammation (Fuchs et al., 2023). The coexistence of these contrasting immune profiles may lead to partial immune modulation without producing measurable changes in pathogen load or antigen expression (Easton et al., 2020; García-Bernalt Diego et al., 2025). This interpretation is further supported by emerging evidence that helminth infections can reshape the gut microbiota and alter bacterial colonization dynamics, although such effects do not necessarily translate into detectable differences in bacterial burden (Wang et al., 2023).

Alterations in hematological and biochemical parameters are common in infectious diseases and serve as important diagnostic and prognostic indicators. In the present study, we examined changes in blood cell profiles and serum proteins in patients infected with *S. mansoni*, *H. pylori*, and both. A significant elevation in neutrophil percentages was observed among *H. pylori* mono-infected patients, a pattern that persisted in co-infected individuals without further significant increase. This neutrophilia likely reflects localized micro-inflammatory reactions induced by *H. pylori*, mediated through proinflammatory cytokines such as TNF, IL-1, IL-6, IL-8, and IL-17A, which stimulate leukocyte production and differentiation (Kim, 2023). Neutrophil lifespan may also be prolonged by *H. pylori*-derived neutrophil-activating protein (HP-NAP) (Cappon et al., 2010). In contrast, eosinophilia was prominent in *S. mansoni*-infected patients regardless of *H. pylori* co-infection. Eosinophilia is a well-recognized hallmark of helminth infections, reflecting Th2-skewed immune responses driven by repetitive and prolonged antigen exposure (Huang and Appleton, 2016). Parasite-specific T lymphocytes produce IL-5, IL-4, and IL-13, which promote eosinophil proliferation, recruitment, and survival (Mitre and Klion, 2021). Tissue granulomatous responses to schistosome eggs rather than the worms themselves are believed to be the principal stimulus for eosinophil accumulation, likely through proteolytic egg-derived enzymes that activate local inflammatory pathways (Davies and McKerrow, 2000). Recent work demonstrates active eosinophil recruitment into schistosome granulomas and their persistence during chronic infection (Huang and Appleton, 2016; Mitre and Klion, 2021). Platelet counts were significantly reduced in all infected groups compared with controls, indicating a consistent infection-associated thrombocytopenic effect. In schistosomiasis, platelets can adhere to and kill migratory schistosomula, and both in vivo and in vitro studies show platelet-parasite interactions that may influence host defense (Abdelfattah et al., 2024; Greenman et al., 2025). Experimental models have demonstrated moderate thrombocytopenia following percutaneous cercarial infection, suggesting that platelet reduction may represent a parasite-driven strategy to evade platelet-mediated immune mechanisms (Abdul-Ghani and Hassan, 2010). Emerging evidence also highlights qualitative platelet abnormalities in schistosomiasis, reinforcing their multifaceted dysfunction (Abdelfattah et al., 2024). Similarly, *H. pylori* infection was associated with reduced platelet counts, consistent with reports of immune thrombocytopenia (ITP) in *H. pylori*-infected patients and platelet recovery after bacterial eradication, possibly due to cross-reactivity between antibodies targeting *H. pylori* proteins and platelet surface antigens (Lee et al., 2020).

Red cell parameters, including hemoglobin, hematocrit, RBC count, and indices, were comparable across the four study groups, with no statistically significant differences. Although anemia is frequently associated with both *H. pylori* infection and schistosomiasis, its absence in this cohort may reflect the predominance of low-intensity *S. mansoni* infections and heterogeneity in *H. pylori*-associated iron dysregulation. Recent meta-analyses indicate that anemia and hematologic abnormalities are more strongly linked to moderate or heavy schistosome infections, whereas light infections may exert minimal effects on hemoglobin and red cell indices (Gebrehana et al., 2024). Proposed mechanisms for anemia in *H. pylori* infection, including reduced gastric acid and iron absorption, altered iron transporter expression, and bacterial sequestration of dietary iron, vary across populations and may not manifest uniformly (Kato et al., 2022). Total leukocyte counts remained within normal limits and did not differ significantly among study groups. Although some studies report leukocytosis in schistosomiasis as part of a cellular defense response and in *H. pylori* infection as a marker of mucosal inflammation, these effects may be subtle or dependent on the chronicity and intensity of infection, particularly in populations with predominantly low parasite burdens (Buonfrate et al., 2025; Jiao et al., 2024). Taken together, the hematological profile observed highlights distinct yet complementary immune signatures of the two infections: eosinophilia, a hallmark of helminth-induced Th2 responses; neutrophilia, reflecting bacterial-driven inflammatory activation; and thrombocytopenia, a shared systemic effect of both pathogens. The absence of significant anemia or leukocytosis in this cohort underscores the influence of infection intensity and chronicity on systemic hematological alterations. These integrated findings provide important insights into the differential immune and hematological impacts of *S. mansoni* and *H. pylori* infections and emphasize the need for context-specific interpretation of laboratory changes in endemic settings.

The increased levels of serum globulin observed in individuals infected with *S. mansoni* reflected ongoing antigenic stimulation, which sustained humoral immune activation. Chronic schistosomiasis was known to lead to polyclonal B-cell activation and elevated immunoglobulin production due to continuous egg deposition and granuloma formation (Torsello et al., 2026). Recent immunological studies confirmed a strong association between helminth infections and hypergammaglobulinemia, particularly in populations with repeated exposure (Douglas et al., 2021; Loke et al., 2022). In contrast, levels of albumin and total protein remained stable across the studied groups, indicating preserved liver synthetic function and an absence of severe protein-losing enteropathy in this cohort. In our study, serum ferritin levels did not differ significantly among the study groups, despite considerable inter-individual variability. This finding indicated that iron storage status was not notably influenced by infection status within this population. Previous studies have linked both *S. mansoni* and *H. pylori* infections to iron deficiency (Butler et al., 2012; Monzón et al., 2013); however, these effects tend to be more pronounced in cases of moderate to heavy infections or in chronic gastric inflammation that impairs iron absorption. Recent meta-analyses have reported that *H. pylori* infection is associated with iron deficiency anemia, particularly in children and populations with prolonged untreated infection, primarily due to impaired gastric acid secretion and reduced iron uptake (Kato et al., 2022). Similarly, iron depletion related to schistosomiasis is more commonly observed in heavy infections that cause intestinal blood loss (Leir et al., 2019). The predominantly low infection intensity observed in this study likely accounted for the lack of significant changes in ferritin levels. Given this low morbidity profile, ultrasonography and histopathological assessments were not included, as such procedures require clinical referral, specialized equipment, and invasive sampling that are not feasible or ethically justified in asymptomatic individuals during community-based field surveys. Accordingly, non-invasive diagnostic tools (Kato-Katz, stool antigen ELISA, CBC, serum biochemistry) were selected to ensure feasibility, participant safety, and appropriate alignment with the study's epidemiological objectives.

Importantly, co-infection did not produce additive or synergistic effects on hematological or biochemical parameters. This supports the interpretation that both pathogens act largely independently in this setting, with limited cross-modulation under conditions of low infection intensity. While experimental evidence suggests that helminth infections may modulate immune responses to bacterial pathogens, such interactions appear context-dependent and may not translate into measurable clinical or laboratory changes in human endemic populations (Bhattacharjee et al., 2019; Chen et al., 2023). Collectively, these findings highlight *S. mansoni* as the dominant driver of systemic immune and hematological alterations, whereas *H. pylori* primarily contributes to localized gastric inflammation with minimal systemic impact in this cohort. The observed patterns emphasize the importance of infection intensity as a key determinant of host systemic response and underscore the need for context-specific interpretation of co-infection dynamics in endemic settings.

## 5 Conclusion

This study demonstrates that *S. mansoni* infection is strongly associated with occupational freshwater exposure and is the primary determinant of systemic hematological and immunological alterations in the studied population. In contrast, *H. pylori* infection shows limited association with environmental exposure and minimal impact on systemic parameters. Although co-infection is common in this endemic setting, it does not significantly modify parasite burden or major hematological and biochemical indices, indicating largely independent host–pathogen interactions under low infection intensity conditions. Key infection-related changes, including eosinophilia, thrombocytopenia, and elevated globulin levels, are primarily driven by *S. mansoni* and reflect a characteristic Th2-skewed immune response. The absence of major anemia or biochemical dysfunction further highlights the mild nature of infections in this cohort. These findings underscore the need for targeted schistosomiasis control strategies focused on reducing freshwater exposure, alongside continued surveillance of co-endemic infections. Further longitudinal studies are warranted to clarify interaction dynamics under higher infection intensities and in populations with greater disease burden.

## CRediT authorship contribution statement

**Ashraf Fawzy Mosa Ahmed:** Writing – review & editing, Writing – original draft, Formal analysis, Data curation, Conceptualization. **Hala Shehata Ali:** Writing – review & editing, Writing – original draft, Formal analysis, Conceptualization. **Nabeel H. Alhussainy:** Writing – review & editing, Writing – original draft, Formal analysis, Data curation. **Hany M. El-Wahsh:** Writing – review & editing, Writing – original draft, Investigation, Formal analysis, Data curation. **Bander Hasan Saleh:** Writing – review & editing, Writing – original draft, Formal analysis, Conceptualization. **Turki Asiri:** Writing – review & editing, Writing – original draft, Formal analysis. **Karem Ibrahem:** Writing – review & editing, Writing – original draft, Supervision, Project administration. **Fadi Baakdah:** Writing – review & editing, Writing – original draft, Validation, Project administration, Funding acquisition. **Haifaa A. Mahjoub:** Writing – review & editing, Writing – original draft, Validation, Project administration, Funding acquisition. **Wesam K. Bakhsh:** Writing – review & editing, Writing – original draft, Formal analysis. **Ala A. Azhari:** Writing – review & editing, Writing – original draft, Validation, Project administration, Funding acquisition. **Hatoon A. Niyazi:** Writing – review & editing, Writing – original draft, Validation, Project administration, Funding acquisition. **Doaa Naguib:** Writing – review & editing, Writing – original draft, Validation, Supervision, Investigation, Formal analysis, Data curation, Conceptualization.

## Funding

This work did not receive any specific grant from funding agencies in the public, commercial, or not-for-profit sectors.

## Declaration of competing interest

The authors declare that they have no known competing financial interests or personal relationships that could have appeared to influence the work reported in this paper.
